# Transforaminal debridement with a posterior-only approach involving placement of an interbody bone graft combined with diseased vertebral fixation for the treatment of thoracic and lumbar tuberculosis: Minimum 5-year follow-up

**DOI:** 10.1097/MD.0000000000020359

**Published:** 2020-05-29

**Authors:** Chen Zhao, Lei Luo, Xiaobing Pu, Liehua Liu, Pei Li, Lichuan Liang, Fei Luo, Tianyong Hou, Fei Dai, Jianzhong Xu, Qiang Zhou

**Affiliations:** a Department of Orthopedics, The Third Affiliated Hospital of Chongqing Medical University (Gener Hospital), Chongqing; b Department of Orthopedic Surgery, No. 4 West China Teaching Hospital, Sichuan University, Chengdu, Sichuan; c Graduate School, Ningxia Medical University, Yinchuan, Ningxia; d Department of Orthopedics, Southwest Hospital, The Army (Third Military) Medical University, Chongqing, China.

**Keywords:** diseased vertebral fixation, long-term follow-up, posterior approach, spinal tuberculosis

## Abstract

The aim of this study was to evaluate the clinical and imaging results of transforaminal debridement with a posterior-only approach involving placement of an interbody bone graft combined with diseased vertebral fixation for the treatment of thoracic and lumbar tuberculosis (TB) with a minimum 5-year follow-up.

Sixty-five patients who presented with active thoracic and lumbar TB between October 2006 and August 2013 were retrospectively analyzed: 20 were thoracic TB (group A), 17 were thoracolumbar TB (group B), and 28 were lumbar TB (group C). The patient data, operating time, blood loss, Visual Analog Scale score, Oswestry Disability Index score, correction of kyphosis, recovery of neurological function, and complications were recorded and analyzed.

The patients were followed for 68.7 ± 17.8 months. The preoperative average Cobb angles of kyphosis in patients in groups A, B, and C significantly decreased from 28.2 ± 11.9°, 30.5 ± 16.9°, and 10.9 ± 8.8° before surgery to 8.0 ± 5.4°, 5.0 ± 4.1°, and –4.4 ± 1.6° (– indicates lordosis) after surgery, respectively. At the final follow-up time, the Cobb angles were 9.2 ± 6.1°, 6.8 ± 10.0°, and -3.7 ± 2.0°, respectively. The postoperative Cobb angles of kyphosis were significantly improved in all groups (*P* < .05). The correction loss angles were larger in groups A and B than in group C (*P* > .05). The operating time, blood loss, and complications were not significantly different between the groups (*P* > .05). Three (4.6%) patients developed unhealed TB during postoperative anti-TB treatment, and 6 patients (9.2%) with TB relapsed after healing from surgery.

The posterior-only approach for the surgical treatment of thoracic and lumbar TB achieved satisfactory outcomes over long-term follow-up. The implantation of pedicle screws in diseased vertebrae reduced the range of fixation, but patients with thoracic and thoracolumbar TB should undergo fixation to at least 1 adjacent normal segment. There were some cases of recurrence after TB healed, and long-term follow-up is therefore necessary.

## 1 Introduction

According to the global tuberculosis (TB) report of the World Health Organization in 2018, TB is 1 of the top 10 causes of death and the leading cause of single infectious disease. In 2017, an estimated 10 million cases of single infectious disease were caused by TB worldwide, and the second highest number (9%) of cases was reported in China.^[1]^

Musculoskeletal TB is the most common type of extrapulmonary TB, and spinal TB accounts for approximately 50% of all cases. Although most cases of spinal TB can be cured with conservative treatment, some patients still require surgical treatment because early diagnosis is difficult. The surgical options for thoracic and lumbar TB remain controversial. The types of surgical strategies include an anterior-only approach, a combined anterior and posterior approach and a posterior-only approach.

The anterior-only and combined anterior and posterior approaches can clear lesions under direct visual guidance to promote healing in TB, improve nerve function and correct deformities.^[2–4]^ However, these approaches have some disadvantages, such as increased trauma and complications.^[5,6]^ Reports have described the use of the posterior-only approach in the treatment of patients with minor spinal TB.^[7]^ With the development of these technologies, this approach has been gradually accepted by surgeons.^[8–10]^ However, few reports have included long-term follow-up results.

In this study, we retrospectively analyzed 65 patients with active thoracic and lumbar TB treated with the posterior-only approach involving transforaminal debridement and placement of an interbody bone graft combined with diseased vertebral fixation with the aim of evaluating clinical and imaging outcomes over long-term follow-up.

## 2 Methods

### 2.1 General information

This study is a retrospective study and has been approved by the Hospital Ethics Committee. Between October 2006 and August 2013, 65 patients with active thoracic and lumbar TB underwent transforaminal debridement with a posterior-only approach and placement of an interbody bone graft combined with diseased vertebral fixation in our spine center. The surgical indications included vertebral collapse >50%; local kyphosis of the thoracic or thoracolumbar segment >30°, lumbar segment > 10°, or progressive kyphosis; spinal instability; neurologic impairment caused by spinal cord compression; and the disease condition continued to progress under standard anti-TB treatment. The patients were divided into 3 groups: 20 patients with thoracic TB (group A, range of lesions from T1 to 10), 17 with thoracolumbar TB (group B, T11–L2), and 28 with lumbar TB (group C, L3–S1). The average ages of the patients in groups A, B, and C were 35.6 ± 11.1, 33.9 ± 12.5, and 39.5 ± 12.3 years, respectively. All patients had lower back pain and symptoms of TB, including low fever, night sweats, and emaciation.

### 2.2 Preoperative preparation

Patients with contagious pulmonary TB were first required to go to a specialist hospital for treatment. Routine preoperative examinations were performed in all patients, such as blood routine, stool routine, urinalysis, liver and kidney function, coagulation function, electrocardiogram, chest radiograph, lung function, arterial blood gas analysis, antibody of infectious disease (HBV, HIV, HCV, TP), erythrocyte sedimentation rate (ESR), C-reactive protein (CRP). Then, X-ray, computed tomography, and magnetic resonance imaging (MRI) were used to identify the lesion location and spinal stability. Patients received treatment involving nutritional support to correct anemia and hypoalbuminemia, and all were administered anti-TB drugs for at least 2 weeks before the operation. The chemotherapy regimen involved isoniazid (300 mg, per os [PO], every day [QD]), rifampin (450 mg, PO, QD), ethambutol (750 mg, PO, QD), and pyrazinamide (500 mg, PO, 3 times daily). The patients also received levofloxacin (200 mg, intravenously, twice daily) during hospitalization. Surgical treatment was performed when symptoms of TB were relieved, the ESR and CRP level decreased, and anemia and hypoalbuminemia were corrected. If neurologic impairment was progressive, early surgery was necessary.

### 2.3 Surgical technique

A midline posterior approach was used to expose the bilateral lamina and facet joint. Bilateral or unilateral facet joints and transverse processes were removed. The TB lesions were completely debrided through transforaminal and paravertebral approaches. The pedicles of the diseased vertebrae were retained if there were no TB lesions. Screws were implanted in diseased vertebrae if possible. When screws were implanted in the bilateral pedicles of diseased vertebrae and when >50% of the vertebrae remained, thoracic and thoracolumbar cases were fixed to 1 upper and 1 lower normal segment, while lumbar cases were fixed to only the lesion segment. Otherwise, the range of fixation extended to an additional segment. Then, a polyether ether ketone cage or titanium mesh full of autogenous bone was used to perform interbody fusion. The polyether ether ketone cage was used in cases with small bone defects, while titanium mesh was used in cases with large bone defects. The lamina defect area was covered by slice autologous or allograft cortical bone which were constructed according to the size of the defect. Then, the bilateral posterolateral fusion is performed. An extraperitoneal anterior approach was performed to address psoas or iliac abscesses during the same surgery.

### 2.4 Postoperative management

Patients received postoperative treatment with anti-infection and nutritional support. The drainage tube was removed when the volume of drainage was <10 mL/24 h. During the first week after the operation, patients were encouraged to ambulate while wearing a brace. The total time required to wear the brace was 3 to 6 months. Anti-TB treatment lasted 12 to 18 months, and the treatment regimens were the same as those applied preoperatively. All patients were followed-up at 3, 6, 12, 18, and 24 months after surgery and then once a year thereafter. When bone fusion was observed on computed tomography, when there were no remaining TB lesions on MRI, and when ESR and CRP levels were normal, we considered the TB to be healed.

### 2.5 Evaluation and statistical analysis

The characteristics of the patients, operating time, blood loss, Visual Analog Scale (VAS) score, Oswestry Disability Index (ODI) score, kyphosis angle, neurofunctional improvement, and complications were recorded. The kyphosis angle was measured by the Cobb angle, and we defined kyphosis as a lumbar angle >0° and thoracic and thoracolumbar angles >10°. Neural function was assessed based on the American Spinal Injury Association impairment scale.

The data were analyzed using IBM SPSS Statistics Version 19. Operating time, blood loss, VAS score, ODI score, and kyphosis angle were analyzed and compared using analysis of variance. Patient characteristics and complications were analyzed using the Chi-squared test.

## 3 Results

The mean follow-up time was 68.7 ± 17.8 months (range 60–130 months). The patient data are shown in Table 1. Multiple segments (more than 1 segment) and kyphosis were more likely to be involved in patients in group A (35%, 75%) and group B (11.8%, 58.8%) than in those in group C (7.1%, 14.3%). Psoas or iliac abscesses were more likely to occur in groups B (35.3%) and C (32.1%) than in group A (5%). There was no significant difference in age, sex or neurological impairment among the groups.

**Table 1:**
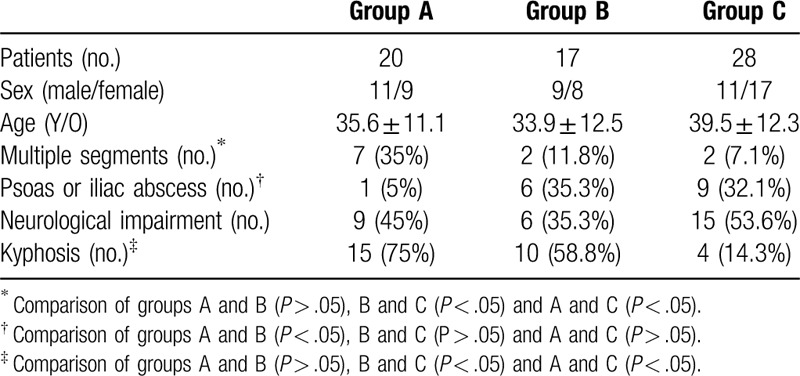
Patient data.

At the final postoperative follow-up, VAS and ODI scores had significantly improved in all 3 groups. There was no significant difference in operating time, blood loss, VAS score, or ODI score among the 3 groups. The detailed clinical outcomes are shown in Table 2.

**Table 2:**
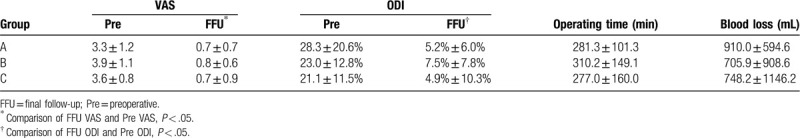
Clinical outcomes.

The average Cobb angles of the patients with kyphosis in groups A, B, and C were 28.2 ± 11.9°, 30.5 ± 16.9°, and 10.9 ± 8.8°, respectively. The preoperative Cobb angle of kyphosis was larger in groups A and B than in group C and significantly decreased postoperatively to 8.0 ± 5.4°, 5.0 ± 4.1°, and –4.4 ± 1.6° (the negative number indicates lordosis), respectively. At the final follow-up time, the Cobb angles were 9.2 ± 6.1°, 6.8 ± 10.0°, and –3.7 ± 2.0°, respectively. The correction loss angle was larger in groups A and B than in group C. The correction of kyphosis is shown in Table 3.

**Table 3:**
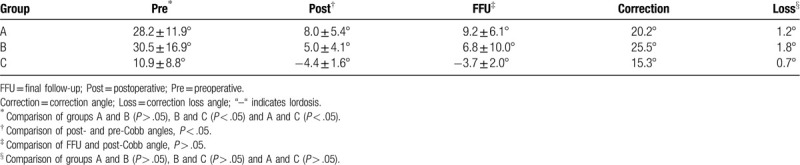
Correction of kyphosis (Cobb angle).

**Figure 1:**
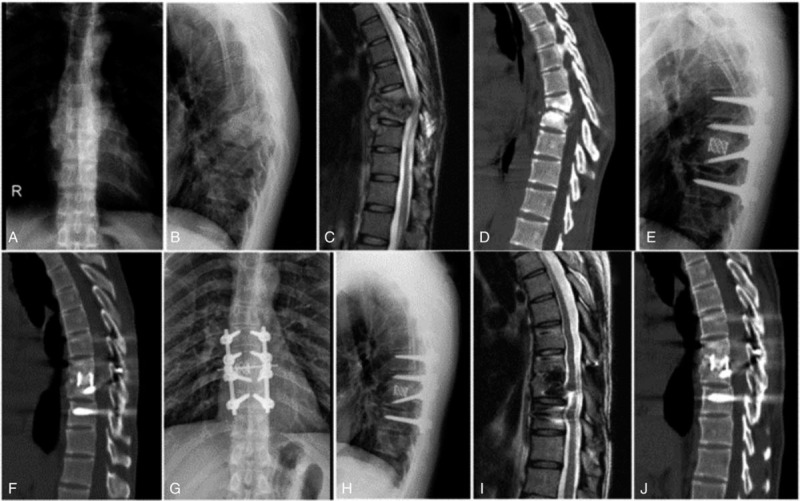
Case I: A 31-year-old male patient with T8–9 tuberculosis in group A. A–D show the preoperative X-ray, computed tomography (CT), and magnetic resonance imaging scans. Screws were implanted in the bilateral pedicles of diseased vertebrae, and the remaining vertebrae were >50% maintained; thus, the range of fixation was from 1 upper to 1 lower normal segment. E and F show the 1-year postoperative X-ray and CT scans, respectively. G–J show the 4-year postoperative X-ray, CT, and magnetic resonance imaging scans, as indicated.

**Figure 2:**
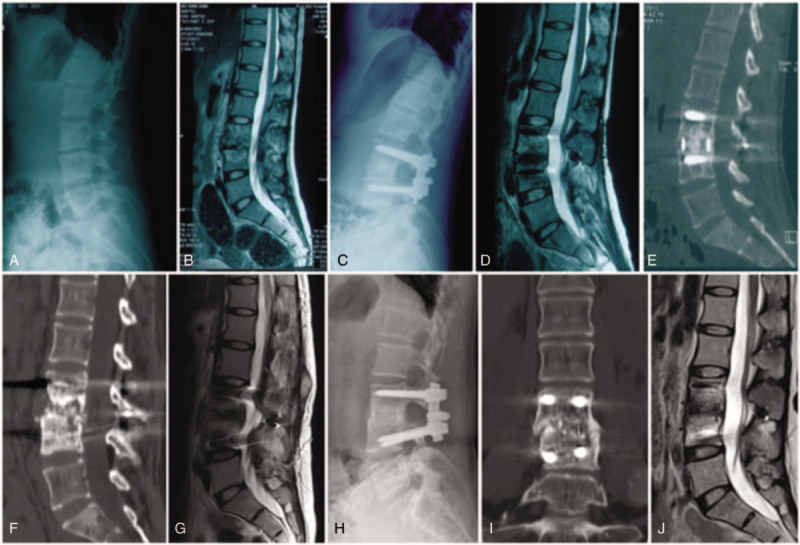
Case II: A 24-year-old female patient with L3–4 tuberculosis (TB) in group C. A and B show the preoperative X-ray and magnetic resonance imaging scans, respectively. Screws were implanted in the bilateral pedicles of diseased vertebrae, and the remaining vertebrae were >50% maintained; therefore, the range of fixation was only the lesion segment. C–E show that TB was cured 1 year after surgery. F and G show TB relapse 3 years after surgery. H–J show that TB was cured by anti-TB treatment again at 5 years after surgery.

The complications that occurred in each group are shown in Table 4. Three (4.6%) of the 65 patients developed unhealed TB during postoperative anti-TB treatment, and the other patients achieved bony fusion. Six patients (9.2%) experienced TB relapse after healing. Two of 3 patients with unhealed TB were cured with a posterior-only reoperation, and the other was cured with conservative treatment. In group A, 1 patient experienced TB relapse 72 months after surgery. In group B, 2 patients with TB relapsed 59 and 63 months after surgery. In group C, 3 patients with TB relapsed 23, 35, and 48 months after surgery. The patient in group C who experienced TB relapse at 23 months was cured with a posterior-only reoperation, while the others were cured with conservative treatment. In group C, the lumbar artery was injured during the operation in 1 case but was cured after embolization. All other complications were cured after conservative treatment. There was no significant difference in the incidence of complications among the 3 groups Figs. 1 and 2.

At the final follow-up time, all patients with neurological impairment had improved by at least 1 level on the American Spinal Injury Association impairment scale. Each patient with grade B and C impairment in group A improved to grade D, while the other patients in the 3 groups improved to grade E.

## 4 Discussion

Transforaminal debridement with a posterior-only approach and placement of an interbody bone graft combined with fixation is a new surgical method for thoracic and lumbar TB. There is some controversy regarding whether posterior-only surgery can be used to effectively debride TB lesions as the operation involves a non-direct view and destabilization of the spine with destruction of the posterior spinal column.

Debridement methods with a posterior-only approach mainly include transpedicular and transforaminal approaches. Zhang et al reported that debridement with a posterior transpedicular approach combined with placement of a bone graft and fixation could heal TB and improve neurological function.^[11]^ Wang et al resected the spinous process and unilateral facet joint to debride TB lesions at 270°.^[6]^ In this study, we debrided TB lesions using a transforaminal approach, and more than 95% of the patients were cured. Debridement with a bilateral transforaminal approach could remove TB lesions effectively at 360°, even though some operations were performed with a non-direct view. This method is convenient for spinal canal decompression and allows spinous processes and ligaments to be maintained. In addition, preservation of the pedicle provides a condition allowing the implantation of screws. Cerebrospinal fluid leakage can easily occur during posterior surgery but does not cause serious consequences, such as TB meningitis. Performing the operation under a non-direct view may result in damage to the anterior vessels, and the relationship between vessels and lesions should therefore be observed with MRI before surgery.

Spinal TB destroys the anterior column in most cases, and the collapse of the anterior column causes kyphosis.^[12]^ Kyphosis correction and spinal stability are important factors in spinal TB surgery. A meta-analysis reported that posterior surgery was better than anterior surgery for correcting kyphosis.^[13]^ Our study demonstrates that posterior surgery can correct kyphosis and restore lumbar lordosis. The pedicle screw system used in posterior surgery can provide spinal 3-column stability. Anterior column support combined with posterior column compression and osteotomy can, when necessary, effectively correct kyphosis.

To improve the stability of the spine, most surgeons adopt a fixation strategy involving fixation to 1 or 2 adjacent normal segments to treat thoracic and lumbar TB.^[4,11,14,15]^ However, this strategy sacrifices normal spinal motion. Some studies have reported that pedicles can provide approximately 70% of the holding force, and the implantation of pedicle screws in fractured vertebrae can reduce screw stress while increasing the axial load and providing anti-flexion and anti-rotation abilities to the entire internal fixation system.^[16,17]^ In many cases, TB destroys the vertebral body but not the pedicle, providing conditions allowing the implantation of screws. We believe that pedicle screws can provide great stability when they are implanted in the bilateral pedicles of diseased vertebrae and in cases where >50% of the vertebrae remain, thus appropriately reducing the range of fixation. This study shows that this fixation strategy can effectively stabilize the spine. The correction loss angle was only 0.7° to 1.8°.

The loss angle was larger in groups A and B than in group C. Pedicle screws placed in the lumbar region provide strong stability because of the large pedicles and vertebral bodies. Thus, the lumbar cases were fixed only at the lesion segment to allow more spinal motion when possible. However, the pedicles and vertebral bodies of the thoracic and thoracolumbar regions are small, and the thoracolumbar region is the site of stress concentration; thus, the range of fixation requires at least 1 adjacent normal segment. The screws should be implanted on the anterior edge of the vertebral body in an upper or lower direction to avoid the bone defect area and increase the holding force.

Unhealed or relapsing spinal TB is a serious complication. Wang et al reported that the postoperative recurrence rate of spinal TB was 3.2%, while that of complex TB was as high as 24.1%.^[18]^ Ren et al reported an unhealed or relapse rate of 10.3% in a 28.7-month follow-up study.^[19]^ Many short-term follow-up studies have reported satisfactory outcomes and a low recurrence rate following posterior surgical treatment of spinal TB.^[3,15,20]^ However, few reports have included long-term follow-up results. In this study, 4.6% of patients developed unhealed TB during postoperative anti-TB treatment, while 9.2% of patients developed relapse after healing, with the latest case occurring 72 months after surgery. Postoperative unhealed TB may be related to incomplete removal of lesions, nonstandard chemotherapy, drug resistance, hypoimmunity, and so on. TB relapse after healing may be associated with resting-stage mycobacterium TB recurrence when patient immunity is low. Therefore, long-term follow-up is necessary in patients with thoracic and lumbar TB.

Thoracic, thoracolumbar and lumbar TB have different characteristics due to anatomical and biomechanical differences. Lumbar TB can compensate for some kyphotic deformities due to physiologic lordosis and large discs.^[21]^ The results of this study also show that the angle and incidence of kyphosis were significantly lower in group C than in groups A and B. The lesions easily spread through the interspace of the psoas major and formed gravitation abscesses in thoracolumbar and lumbar TB. Debridement with an anterior extraperitoneal approach was required if the gravitation abscess could not be removed with posterior-only surgery.

Of course, this study has some limitations, such as a single-center retrospective study and the inadequacy number of cases studied. The results of this study need to be further confirmed by studies with multi-center, prospective and more cases.

## 5 Conclusions

The surgical treatment of thoracic and lumbar TB with a posterior-only approach can achieve satisfactory outcomes over long-term follow-up. Transforaminal debridement can preserve the pedicles, and the implantation of pedicle screws in diseased vertebrae can reduce the range of fixation. However, patients with thoracic and thoracolumbar TB should undergo fixation to at least 1 adjacent normal segment. Cases of recurrence may still occur after TB healing, and long-term follow-up is necessary.

## Author contributions

**Conceptualization:** Xiaobing Pu, Qiang Zhou.

**Data curation:** Chen Zhao, Liehua Liu, Pei Li, Lichuan Liang, Fei Luo, Tianyong Hou, Fei Dai, Jianzhong Xu, Qiang Zhou.

**Formal analysis:** Chen Zhao, Liehua Liu, Qiang Zhou.

**Investigation:** Chen Zhao, Lei Luo, Xiaobing Pu, Liehua Liu, Pei Li, Lichuan Liang, Fei Luo, Tianyong Hou, Fei Dai, Jianzhong Xu, Qiang Zhou.

**Methodology:** Chen Zhao, Lei Luo, Xiaobing Pu, Qiang Zhou.

**Project administration:** Lei Luo, Xiaobing Pu, Qiang Zhou.

**Software:** Chen Zhao.

**Supervision:** Qiang Zhou.

**Writing – original draft:** Chen Zhao.

**Writing – review & editing:** Chen Zhao, Liehua Liu, Pei Li, Qiang Zhou.
